# Assessing *Sus scrofa* diversity among continental United States, and Pacific islands populations using molecular markers from a gene banks collection

**DOI:** 10.1038/s41598-019-39309-9

**Published:** 2019-02-28

**Authors:** D. A. Faria, C. Wilson, Samuel Paiva, H. D. Blackburn

**Affiliations:** 1 Present Address: Departamento de Ciências Fisiológicas, Instituto de Biologia, Campus Darcy Ribeiro, Universidade de Brasilia, Asa Norte, Brasilia, DF 70910-900 Brazil; 2 National Animal Germplasm Program, National Laboratory for Genetic Resources Preservation ARS-USDA, Fort Collins, CO 80521 USA; 3 Present Address: Embrapa Recursos Genéticos e Biotecnologia, Laboratório Genética Animal, Final Avenida W5 Norte, Brasília, DF 70770-917 Brazil

## Abstract

Human migration and trade facilitated domesticated livestock movement, gene flow and development of diverse populations upon which agriculture is based. In addition, varying USA ecological conditions has led to a diverse set of livestock populations to utilize. Quantifying genetic diversity of these populations is incomplete. This paper quantifies genetic diversity captured by the National Animal Germplasm Program and explores genetic structure and differences among 19 pig populations (feral populations from Pacific islands, continental US, and Chinese breeds) using 70,231 SNP from 500 animal samples. Among continental US breeds F_is_ was consistently low suggesting genetic variability is sufficiently available for breeders to use. A unique population structure using principal component analysis illustrated clear distinctions between Duroc, Yorkshire, Hampshire, breeds of Chinese origin, and feral Pacific Island populations were identified. Five Y chromosome haplotypes were evaluated and demonstrated migration patterns from European, central Asia, and potentially Polynesian waves of gene flow. Quantifying diversity and potential origin of Pacific populations provides insight for future uses, and the need for preservation. Viewing gene bank holdings in context of diversity measures we found a lack of inbreeding within breeds, suggesting the collection represents a wide sampling of individual breeds.

## Introduction

The objective of this study was to quantify the genetic diversity of a substantial number of pig breeds found in the United States (US) as previous assessments tend to be focused on Europe and Asia^1–4^. Specifically the assessment provides insights to the genetic variability captured in the USDA’s swine germplasm collection^5,6^. The study evaluated major breeds, often termed international commercial breeds, rare breeds, Chinese breeds imported into the U.S. during the late 1980’s, and feral populations from the Pacific islands of Hawaii, Kauai, and Guam. It has been hypothesized that such feral populations may be harbingers of genetic diversity not found in other US populations given the potential for either Asian or Polynesian ancestry^7^.

Current feral pig populations on the Hawaii and Kauai are the product of a migration wave associated with Polynesian settlers followed by European explorers during the 1700’s^7,8^ and additional domestic pigs gone feral upon the chain of Hawaiian Islands. McCann *et al*.^8^ suggested animals sampled in the Hawaiian Islands originated from Europe, China, Papua-New Guinea, Vanuatu, and shared haplotypes with other domestic-feral pigs and *S*. *verrucosus* (from Java). Larson *et al*.^9^ suggest Hawaiian Islands pigs as members of the Pacific clade. No analysis has been performed on Guam’s feral population. But as part of the Mariana island chain, feral pigs might belong to an East Asia clade^9^.

Breed development occurred in three geographic areas^10^: England, China, and the U.S. Obviously, U.S. breeds were imported or derived from other parts of the world at varying points in time; however, the imports have been used to maintain the original breed or construct new breeds like Duroc, Hereford, Ossabaw Island, Spots/Poland China, and Chester White. The Duroc in particular is an interesting example as its foundation breeds have not been shown to have strong genetic linkages with other European breeds^1,3^. These authors have suggested that the breed may have been comprised, in part, of Guinea Hog from Africa (among other breeds) due to its red color.

A better understanding of the genetic diversity among these U.S. populations has a number of uses, including: general management of genetic diversity by public and private sectors, targeting future germplasm collections, and providing input into policies concerning feral pig populations on Pacific islands.

## Results

### Genetic Diversity

Basic marker statistics of the GGP Porcine HD v1 (57,668 SNPs) have shown that more than half of the markers were polymorphic for feral and rare breeds/genetic groups (Table S1) suggesting the GGP Porcine HD v1 has utility in evaluating pig populations not considered in its development. The Mangalitsa (MA) and Chinese populations had the lowest number of polymorphic SNPs, but the MA and Chinese populations had small sample sizes and/or a genetic bottleneck due to the limited importation. Despite small sample size of the other minor breeds, their average heterozygosity was similar to commercial breed estimates.

The calculated genetic diversity parameters (Table 1) were performed using a smaller SNP panel (8,764) and were uniform among the commercial breeds. Observed heterozygosity for Yorkshire (YK), Landrace (LA), and Duroc (DU) were 0.375, 0.397 and 0.372, respectively. The minor breeds Hereford and Tamworth had similar levels of heterozygosity as the commercial breeds. While other minor breeds may have had higher levels of heterozygosity their limited sample size makes any conclusion problematic. The Pacific islands populations and Chinese breeds had similar observed and expected heterozygosity as the commercial US breeds.

**Table 1:** Sampling information and genetic diversity parameters of 19 pig populations.

Breed/Population	Code	N	Category	Country	Ho	He	F_IS_	N_SNP_
Duroc	DU	65	Commercial	United States	0.372	0.370	−0.003	7905
Berkshire	BE	43	Commercial	United Kingdom	0.335	0.337	0.009	8216
Hereford	HF	22	Minor	United States	0.370	0.359	−0.023	7757
Chester White	CW	27	Commercial	United States	0.370	0.364	−0.015	8415
Hampshire	HS	38	Commercial	United Kingdom	0.351	0.341	−0.024	7627
Yorkshire	YK	106	Commercial	United Kingdom	0.375	0.373	−0.003	8632
Landrace	LA	35	Commercial	Denmark	0.397	0.382	−0.034	7849
Mangalitsa	MA	2	Minor	Austria-Hungary	0.620	0.571	−0.079	3372
Hawaii island	HI	19	Feral	Hawaii	0.322	0.353	0.081	2870
Kauai island	KI	15	Feral	Hawaii	0.365	0.356	−0.017	2192
Spotted	SP	10	Commercial	United States	0.382	0.382	−0.003	7715
Large Black	LB	3	Minor	United Kingdom	0.484	0.475	−0.039	4597
Ossabaw Island	OI	5	Minor	Spain	0.449	0.416	−0.055	5312
Tamworth	TA	10	Minor	United Kingdom	0.403	0.380	−0.053	7582
Guinea Hog	GH	4	Minor	West Africa	0.500	0.441	−0.096	4337
Guam island	GI	6	Feral	Guam	0.375	0.389	0.011	2614
Meishan	ME	52	Minor	China	0.294	0.309	0.031	5726
Fengjing	FE	19	Minor	China	0.374	0.339	−0.056	5439
Minzhu	MI	19	Minor	China	0.352	0.343	−0.015	7782

N: number of samples genotyped; Ho Observed Heterozygosity; He: Expected Heterozygosity; F_IS_: Fixation Index; and N_SNP_: number of polymorphic SNPs with MAF > 0.05 within 8,764 subset.

Overall, the levels of inbreeding in most of the populations evaluated were low indicating the high genetic diversity levels among the *in-situ* population captured in the repository by the germplasm conservation program. The feral population on HI had the highest inbreeding level (0.081) which is higher than the inbreeding levels for KI (−0.017) and GI (0.011), which are geographically smaller islands.

Population differentiation, as measured by pairwise F_ST_, was non-significant for only three comparisons MA vs LB, MA vs OI, and MA vs GH (Table 2). Highest F_ST_ levels were associated with rare breeds, Pacific island, and Chinese populations and the lowest F_ST_ values were found among the CW, YK, and LA, along with HE and DU comparisons (Table 2).

**Table 2:** Pairwise F_ST_ values between 19 pig populations^a^.

	DU	MA	ME	HI	SP	FE	LB	OI	TA	MI	BE	KI	HS	YK	GH	HE	CW	GI	LA
DU	0																		
MA	0.21	0																	
ME	0.25	0.44	0																
HI	0.14	0.24	0.24	0															
SP	0.14	0.20	0.32	0.15	0														
FE	0.23	0.42	0.18	0.22	0.29	0													
LB	0.19	**0**.**34**^**NS**^	0.39	0.22	0.19	0.37	0												
OI	0.20	**0**.**33**^**NS**^	0.39	0.23	0.21	0.37	0,31	0											
TA	0.11	0.22	0.32	0.16	0.13	0.29	0,20	0,23	0										
MI	0.14	0.25	0.18	0.12	0.16	0.16	0,21	0,23	0,17	0									
BE	0.14	0.21	0.27	0.14	0.12	0.25	0,20	0,21	0,15	0,15	0								
KI	0.17	0.32	0.29	0.13	0.20	0.28	0,29	0,29	0,21	0,17	0,18	0							
HS	0.15	0.24	0.30	0.16	0.15	0.27	0,23	0,24	0,17	0,18	0,15	0,21	0						
YK	0.10	0.17	0.21	0.10	0.10	0.18	0,15	0,17	0,11	0,11	0,11	0,13	0,13	0					
GH	0.19	**0**.**34**^**NS**^	0.39	0.22	0.19	0.37	0,32	0,31	0,21	0,22	0,19	0,29	0,22	0,16	0				
HF	0.09	0.23	0.30	0.16	0.15	0.27	0,22	0,23	0,14	0,17	0,15	0,20	0,17	0,12	0,21	0			
CW	0.12	0.18	0.27	0.12	0.11	0.23	0,17	0,19	0,12	0,13	0,12	0,16	0,14	0,08	0,17	0,13	0		
GI	0.16	0.38	0.29	0.16	0.20	0.28	0,32	0,32	0,21	0,15	0,18	0,22	0,21	0,13	0,33	0,19	0,15	0	
LA	0.12	0.17	0,26	0.11	0,11	0.22	0,16	0,18	0,12	0,13	0,12	0,15	0,14	0,08	0,17	0,13	0,07	0.14	0

^a^*P*-value < 0.05.

^NS^not significant.

### Genetic Structure

**Figure 1:**
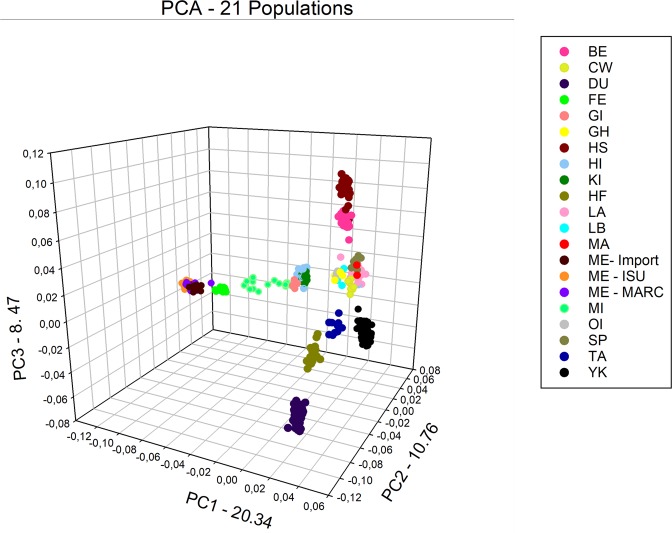
Population structure of all US, Pacific Islands, and China populations analyzed revealed by Principal component analysis. The graph represents a 3D scatterplot of the 3 firsts PCAs, explaining 20.34, 10.76, and 8.47% of the genetic variance among the populations, respectively. BE – Berkshire, CW – Chester White, DU – Duroc, FE – Fengjing, GI – Guam Island, GH – Guinea Hog, HS – Hampshire, HI – Hawaii island, KI – Kauai island, HF – Hereford, LA – Landrace, LB – Large Black, MA – Mangalitsa, ME – Meishan, MI – Minzhu, OI – Ossabaw Island, SP – Spotted, TA – Tamworth, YK – Yorkshire.

The first three principal components (PC) accounted for 39.6% of the variation (Fig. 1). The primary PC explained 20% of total variance and provided a clear separation of the Chinese, Pacific island, and continental breeds. The second and third PCs partitioned the continental breeds, seemingly based upon skin pigmentation (black, white and red) and function (maternal vs sire breeds). Specifically, the second and third PC placed DU, YK, and HS breeds at extreme positions of the second and third PC axis. In between the extreme PC positions’ there was a large grouping of breeds. Interestingly, the Pacific island populations (HI, KI and GI) were positioned closer to Minzhu (MI) and the other Chinese breeds (ME and FE).

**Figure 2:**
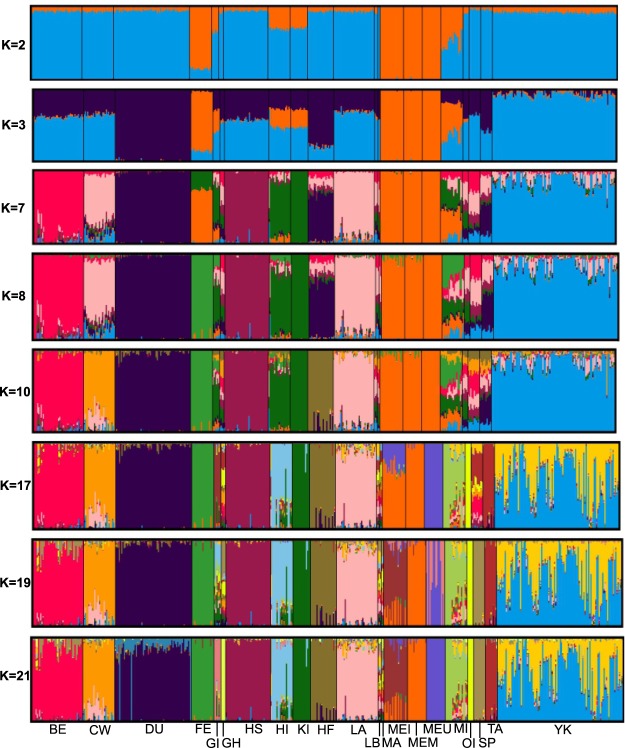
Population Structure of all US, Pacific Islands and China populations revealed by Admixture assignment proportions, where K is the number of assumed ancestral clusters that ranged from 2 to 21. BE – Berkshire, CW – Chester White, DU – Duroc, FE – Fengjing, GI – Guam Island, GH – Guinea Hog, HS – Hampshire, HI – Hawaii island, KI – Kauai island, HF – Hereford, LA – Landrace, LB – Large Black, MA – Mangalitsa, ME – Meishan, MI – Minzhu, OI – Ossabaw Island, SP – Spotted, TA – Tamworth, YK – Yorkshire.

ADMIXTURE was run with K = 2 to 21 and those results were in agreement with PCA. Cross validation error was used to assess which K provided insights into population structure. That said from K = 7 to K = 21 the cross validation curve was asymptotic with an approximate 1.5% change per K. Therefore we selected K of 2, 3, 7, 8, and 10 for evaluation. At K = 2 the breeds were partitioned between Meishan (ME), originating in China, and those from the continental US. Admixed were the populations from GI, HI, KI, MI, and FE (Fig. 2). By K = 3 DU and YK emerged as distinct populations along with ME. For K = 7, 8 and 10 structure among the major breeds, plus HF, KI, HI, ME and FE was evident. At K = 17, ME–U was distinct from ME-I and ME–MARC populations. As larger K’s were evaluated, additional breeds were identified as their own cluster and several breeds were partitioned into subpopulations e.g., YK and ME. The separation of the three ME subpopulations was a function of genetic drift^11^. However, the YK subpopulations are likely due to imported animals during the 1980’s which were widely used within that breed.

**Figure 3:**
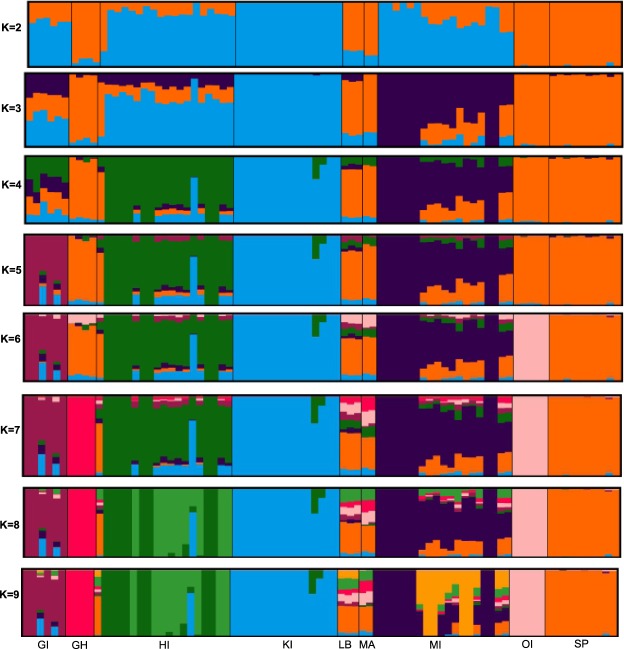
Population Structure of minor, feral, Pacific Islands and China populations revealed by Admixture assignment proportions where K is the number of assumed ancestral clusters that ranged from 2 to 9. GI – Guam island, GH – Guinea Hog, HI – Hawaii island, KI – Kauai island, LB – Large Black, MA – Mangalitsa, MI – Minzhu, OI – Ossabaw island, and, SP – Spotted.

To increase the admixture assessment of the smaller populations’ ADMIXTURE analysis was performed with nine breeds (Fig. 3). K equal four (CV = 0.621) was identified as the most probable number of clusters in this subset of populations. The populations of HI, KI, and MI, plus the grouping of SP, OI, GH, LB, and MA comprised the four clusters. Sample size for the rare breeds was small and may explain the lack of differentiation. However, minor breeds that tend not to be under continuous and breed wide selection can exhibit substantial amounts of admixture, as the GI population does (Fig. 3), similar to the findings with goats^12,13^.

### Y chromosome

**Figure 4:**
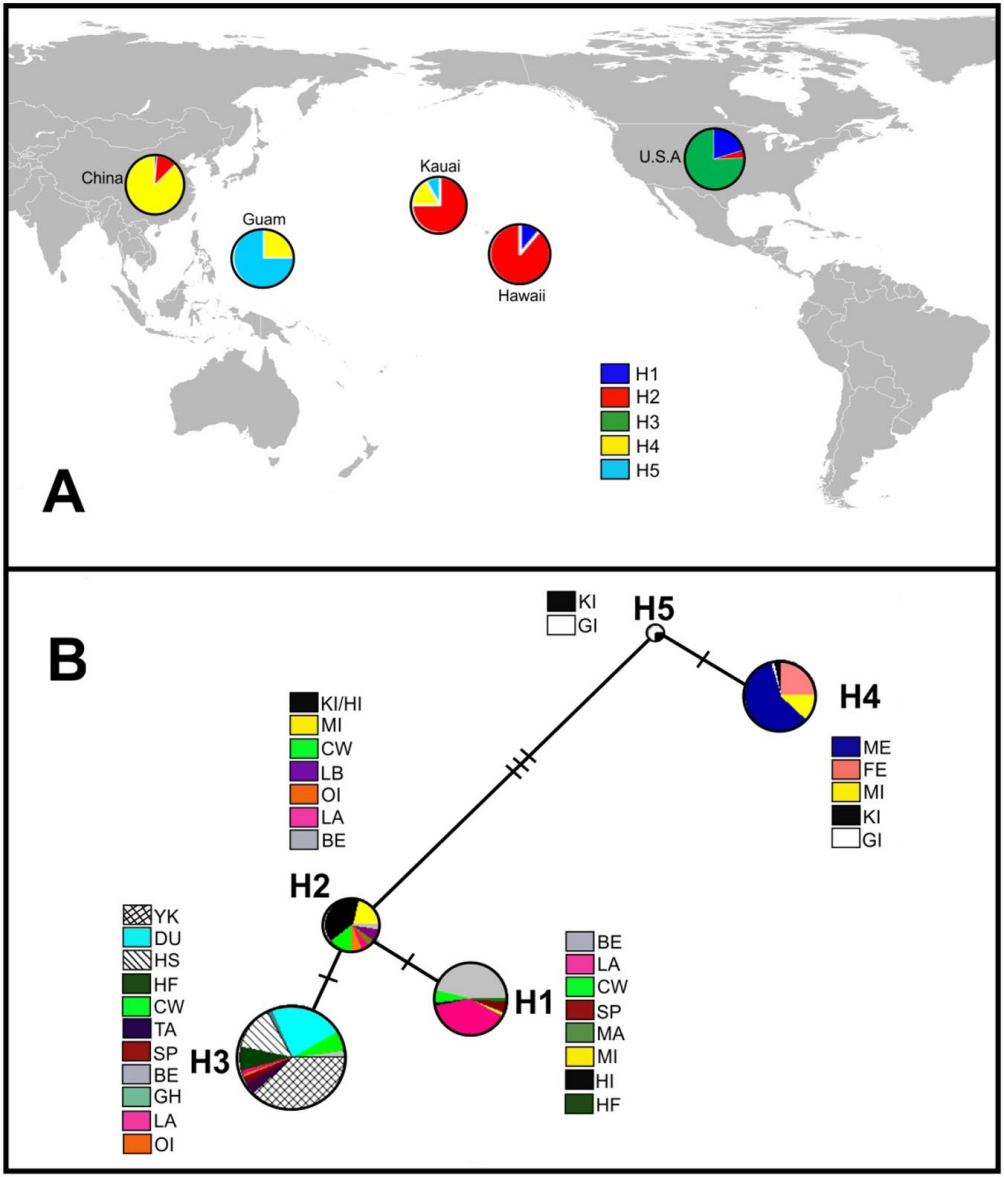
(**A**) Frequency distribution of five Y chromosome haplotypes from sampled pig populations by geographic origin. (**B**) Median-joining network of Y chromosome haplotypes in all US, Pacific Islands and China populations.

Five different haplotypes were found among the animals sampled (Table 3 and Fig. 4). The H3 was present in 278 samples (57%). Except for LB and MA, this haplotype was observed in all breeds that came to the US from Europe. Furthermore, H3 was also the only haplotype observed in DU, YK, TA, HS, and GH breeds. The haplotypes H1 and H2 were present in pigs from the HI and KI, China, and the US mainland. The H4 haplotype was exclusive to animals from China and the Pacific islands. Pigs from Guam and Kauai islands were the only populations exhibiting the H5 haplotype. Median joining network analysis (Fig. 4B) has shown that haplotypes H4 and H5 (China and Pacific islands) are closer than the remaining three haplotypes.

**Table 3:** Absolute haplotype frequency by population for the five haplotypes estimated (H1-H5) using 6 SNPs on the Y chromosome.

Breed	Code	H1	H2	H3	H4	H5	N
Duroc	DU			64			**64**
Berkshire	BE	36	1	6			**43**
Hereford	HF	1		21			**22**
Chester White	CW	4	6	17			**27**
Hampshire	HS			38			**38**
Yorkshire	YK			106			**106**
Landrace	LA	30	2	3			**35**
Mangalitsa	MA		2				**2**
Hawaii island	HI	1	8				**9**
Kauai island	KI		9		2	1	**12**
Spotted	SP	4		6			**10**
Large Black	LB		3				**3**
Ossabaw island	OI		3	2			**5**
Tamworth	TA			10			**10**
Guinea Hog	GH			4			**4**
Guam island	GI				1	3	**4**
Meishan	ME				43		**43**
Fengjing	FE				19		**19**
Minzhu	MI	1	9		9		**19**

N: number of male samples genotyped.

### Effective Population Size

**Figure 5:**
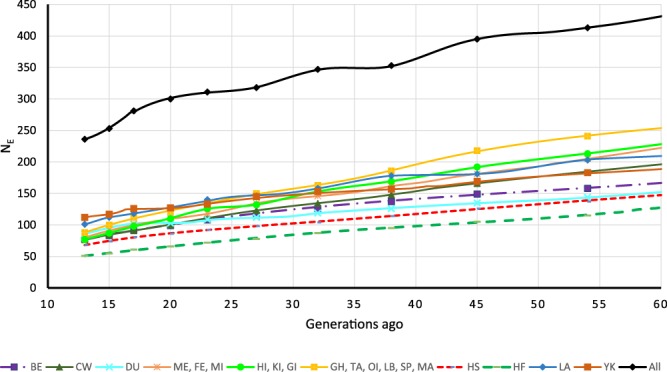
Ancestral and recent effective population size of swine populations place in 10 groups for 60 previous generations. BE – Berkshire, CW – Chester White, DU – Duroc, Chinese (ME – Meishan, MI – Minzhu, FE – Fengjing), Pacific islands (GI – Guam Island, HI – Hawaii island, KI – Kauai island), Minor breeds (GH – Guinea Hog, LB – Large Black, MA – Mangalitsa, OI – Ossabaw Island, SP – Spotted, TA – Tamworth), HF – Hereford, HS – Hampshire, LA – Landrace, YK – Yorkshire.

As most pig breeds were developed after 1800 we report Ne for the tested groups to generation 60 (Fig. 5). The most recent generation had an Ne ranging from 51 to 112 for the tested populations. The Ne trend was linear across generations and the r^2^ decay rates were similar to other reports on swine^14^. All breeds were pooled to compute Ne on a species level, where Ne was estimated to be 236.

## Discussion

The SNP chip used in this study was sufficient to explore genetic diversity and structure among rare and specialized pig breeds originating from Asia and Europe even with expected ascertainment bias. Pigs have a higher linkage disequilibrium than, for example, ruminant species and this was confirmed with the final number of SNP used in the data set of 8,764 markers. By using the GGP Porcine HD v1 chip we had access to SNP located on the Y chromosome which enabled a deeper analysis of genetic variability.

Based upon the population parameters evaluated US breeds, as represented by germplasm samples in the repository, are genetically robust with regard to heterozygosity levels. Genetic diversity of repository samples were similar to European, Asian and previous US values^15–19^. Inbreeding (F_IS_) within breed/population were low suggesting boars sampled for the germplasm collection were lowly related and a broad range of genetic variability has been captured in the gene bank. Furthermore, F_IS_ values were lower than the pedigree based inbreeding levels previously computed^20^ and Canadian values^15^.

Among populations, pairwise F_ST_ suggested the breeds evaluated are distinct; similar to^21^, using microsatellite markers (F_ST_ = 0.10 between DU and YK). As reported in the literature^15^, the estimates of F_ST_ within European and within Asian origin populations are smaller than among Asian and European breeds and are in agreement with ADMIXTURE analysis. Paired combinations of F_st_ between feral populations of the Pacific and breeds originating in China were similar and confirm the PC analysis.

The number of polymorphic SNP in the larger panel (Table S1) and within the 8,764 subset, is lower for the Pacific island populations and minor breeds (approximately one-third the number of SNP in commercial breeds). This may be due to ascertainment bias i.e. the core of the BeadChip was developed based on four commercial breeds (Duroc, Landrace, Large White, and Piétran) and a wild boar population^22^. On the other hand, the low number of markers should not be an issue since these markers did not suggest the presence of bottlenecks (data not shown); conversely, they highlight the high genetic diversity of these populations (high H_o_ and low F_IS_).

The PCA provides a unique view of the relationship among the 19 populations evaluated, in terms of the placement of US mainland breeds (and selection efforts which have differentiated these populations), Pacific islands, and breeds from China (Fig. 1). It suggests feral populations in the Pacific are a confluence of east and west origin and the result of various waves of human migration^7,23^. Typical of PCA analysis a relatively small proportion of genetic variance was accounted for, however previous reports for pigs display similar proportions^18,20,24^. Our results underscore the divergence of Duroc which usually appears as a main and separate cluster from other breeds^18,20,25,26^.

Combining PCA and F_ST_ analysis suggest DU, HS, and YK are genetically distinct from one another. While this might be expected when comparing YK to the two sire breeds we anticipated more similarity between DU and HS. As to the next tier of commercial breeds (Berkshire, Landrace and Chester White) pair-wise F_ST_ values were similar to the DU – HS value. The three maternal white breeds had significant but smaller pair-wise F_ST_ values, the PC analysis indicated the YK was distinct from the LA and CW (both of which were shown to be closely associated; Fig. 1). ADMIXTURE results support the PC analysis for when K = 8, CW and LA were members of the same cluster, by K = 10 they were placed in different clusters while YK had its own cluster by K = 3.

The expansion of genetic structure analysis performed with ADMIXTURE corroborates the common pattern that separates populations of European and Asian origin^1^; while Pacific island populations and MI were substantially admixed. When K = 3 the DU, YK, and ME had separate clusters and the remaining European breeds were admixed. Interestingly at K = 3 DU shows little to no remnant of the Chinese origin breeds, underscoring the breed’s unique genetic composition. Through subsequent clusters in the ADMIXTURE run DU showed little to no admixture, from K = 3 to K = 19, while the YK showed higher levels of admixture, from K = 7 to 21, which may be a function of newly imported Yorkshires that were under different selection strategies or separated by genetic drift. By K = 10 KI was segregated into its own population but HI and GI remained admixed, that said substantial portions of the HI and GI populations were assigned to the KI cluster. Interestingly the MI, OI, and GH all had proportional assignments to the KI cluster underscoring the east - west confluence. At K = 10 we noted that MI, TA, SP, and GH were highly admixed suggesting they are a reservoir of genetic diversity.

Y-chromosome results provide insights into the migration of these swine populations. Interestingly GI’s population had haplotypes from China (H4) and an unknown origin (H5). We hypothesize that H5 may be part of the Pacific clade as reported earlier^9^ and observed in other livestock^23^. If this assumption is correct it suggests the Guam feral population may be the result of two different migrations from east Asia and the south Pacific. The identification of the H4 and H5 haplotypes on KI suggests further eastward migration of swine populations with Polynesian and East Asians into the central Pacific. The presence of the H4 haplotype on KI would be counter to^9^. The remaining haplotypes on KI (H2) and HI (H1 and H2) were present among continental US populations, suggesting admixture with later importation of European derived pigs. The PCA and ADMIXTURE results appear to support the haplotype analysis among the Pacific island populations. In Fig. 1 the Pacific Island populations were intermediate to the Chinese and continental US populations. In the ADMIXTURE analysis the island populations have high proportions of admixture relative to other highly defined clusters.

Haplotypes H1 and H2 are common in the continental US and the Hawaiian Islands. Haplotype-2 was the most cosmopolitan of haplotypes appearing in China, Hawaiian Islands and US. Additionally its internal position in the network analysis (Fig. 4) suggests that H2 might be an ancient haplotype, even though it is more genetically prevalent in US haplotypes. Its presence in minor and rare breeds could support the view of H2 being an older haplotype. If this is correct, its presence on the Hawaiian Islands might be the result of earlier exploration by European’s in the 18^th^ century. However, because of its presence in the Chinese MI breed H2 could have originated during the domestication of pigs in China and imported into Europe during the 18^th^ century.

Haplotype H3 was observed in 11 of the 19 populations but it was only found in the continental US. Also, it was the only haplotype among the prominent breeds: DU, HS and YK; and minor breeds: TA, Hereford and GH. Given that the Chinese and Pacific populations lack the H3 haplotype suggests a European origin, and may have as an original source wild boars found west of the Caucus Mountains^27^.

A major aspect of this study was to provide a first assessment of the within and among breed genetic diversity captured in the national gene bank collection. Effective population sizes for commercial breeds were in agreement with work^14^ assessing Canadian DU, YK, and LA using LD. This study’s results are also in agreement with the Ne computed with pedigree information^19^ for DU, YK, LA, HS, and BE. In all instances Ne was >50 exceeding the minimum Ne suggested by FAO^28^. By pooling study populations we noted substantial variation, as suggested by an Ne = 236 which resides in the gene bank and can be used to advance breeding goals, similar to that reported with sheep^29^.

For breeds with more than 20 animals genotyped the results tended to be homogeneous, in that, levels of heterozygosity and inbreeding were similar and at levels that suggest sufficient genetic variability exists among the populations captured in the repository^28^. Results for breeds with less than 15 animals’ are viewed as preliminary. That said, genetic parameters for SP, HE and TA were similar to breeds with larger sample size. Up to K = 10 in the ADMIXTURE analysis the SP and TA were admixed, at K = 10 the HF were placed in a unique cluster, but TA was not. Those early admixture patterns for low-medium K values reflect clusters of breeds that share recent admixture events, higher genetic diversity and/or were not undergoing selection nor experienced a recent bottleneck^13,15^.

The samples from Guam, Kauai, Hawaii, and US/Chinese ME provide interesting models for evaluating small isolated populations. The ME included randomly bred lines; while the island populations are all feral but confined to the island sampled. Genetic parameters (Table 1) for GI and KI suggest the populations have similar levels of heterozygosity to the numerically larger commercial breeds in the continental US and greater than the ME samples. As the number of ME imported was small, our results suggest larger numbers of East Asian and Polynesian pigs were part of the migration to those islands. Another possible interpretation is that the admixture of the different importations have broadened the genetic base of the feral populations. Island size also supports our admixture conjecture, as Hawaii is approximately 7 times larger than Kauai and 19 times larger than Guam. With Kauai and Guam having potentially multiple waves of immigration plus a potential sex ratio close to 1:1 inbreeding accumulation would be dampened and a relatively high effective population size maintained as demonstrated by the computed Ne of 77. As a result, such populations may serve several important functions in the future. For example, they could serve as sentinel populations for monitoring genetic by environmental interactions in relation to a changing climate or a resource to study a wide variety of diseases with what may be naive populations. The feral population may also serve non-biological functions in relation to cultural practices of indigenous human populations found in the Pacific.

## Conclusions

Large and substantial genetic differences in pig populations dispersed across the continental US, Pacific Islands and Chinese breeds were found. Results suggest that feral populations from Pacific islands have a combination of European, East Asian and potentially South East Asian origin. Identifying the diverse origins of feral pigs on the Pacific islands contributes to the policy dialog about the control/eradication and/or conservation of these populations. While there may be environmental issues, the populations in question are unique and may have cultural significance for persons of Polynesian ancestry. Exploring feral genomes further may reveal other genetic attributes of interest for the research community and industry.

This study’s evaluation of commercial breeds acquired for gene banking purposes is encouraging. Samples from commercial breeds suggest a range of genetic variability has been captured by the gene bank. As an indicator of genetic variability among *in-situ* populations, our results suggest no lack of genetic variability. Some rare breeds showed a lack of genetic distinction in PC analysis and were admixed which was likely due to limited sample size. But, HE and TA were distinct (particularly in PC analysis) and were shown to have measures of genetic variation in the same ranges as the commercial breeds. As the collection of swine genetic resources in the gene bank continues to grow it will be a resource to further explore various aspects of genetic diversity or gene function.

## Material and Methods

### IACUC Review

No samples were collected for this study; rather they were collected as part of other studies or program activities not associated with this study. Semen samples were acquired from swine artificial insemination centers by the National Animal Germplasm Program as part of efforts to conserve genetic resources. Blood samples from Pacific island populations were collected by USDA Wildlife Services in their routine efforts to manage feral populations and then provided to the National Animal Germplasm Program.

### Breeds and Populations

This study focused on assessing populations found upon the continental US and its Pacific islands. We were particularly interested in evaluating feral Pacific populations’ origin and relationship to rare and commercial breeds. Samples and data originated from 19 pig breeds (Table 1) or genetic groups (N = 500): Berkshire, Duroc, Hampshire, Landrace, Yorkshire, Chester White, and Spotted represented commercial breeds; Fengjing, Meishan, Minzhu represented Chinese breeds originally imported to the US in the late 1980’s; Guinea Hog, Hereford, Large Black, Mangalitsa, Ossabaw Island, and Tamworth were among the minor and rare breeds; and feral hogs from the Pacific islands of Guam, Kauai, and Hawaii (Table 1). All breeds, with exception of Mangalitsa, Meishan, Fengjing, and Minzhu were established in the US for one century or longer. Total genomic DNA were extracted from sperm, blood or FTA cards (Guam, Kauai and Hawaii). The protocol varies according to the origin of the biological material and were adjusted by Neogen Genomics (http://genomics.neogen.com/en/). All samples and genotypes are deposited with the USDA ARS National Animal Germplasm Program (https://nrrc.ars.usda.gov/A-GRIN/database_collaboration_page_dev).

The Meishan was composed of three subpopulations^11^ denoted by Meishan – IM (samples from the original Meishan importation), Meishan – U (randomly mated control herd maintained at Iowa State University) and Meishan – MARC (randomly mated control herd maintained by USDA-ARS).

Substantial distances exist between the geographic areas sampled. From continental U.S. to the Hawaiian Islands is approximately 3,900 km and Hawaiian Islands to Guam is approximately 6,000 km. The distance between the Hawaiian Islands of Hawaii and Kauai is approximately 500 km. Island size varies greatly: Guam, 544 km^2^; Kauai, 1,440 km^2^; and Hawaii, 10,432 km^2^. Guam’s samples were from the same area of that island.

### Genotyping and quality control

Samples were genotyped by a commercial vendor (Neogen Genomics, Lincoln, NE), using GGP Porcine HD v1 (70,231 SNP). Final Reports generated with Genome Studio Software (Illumina, San Diego, CA, USA) were imported into SNP & Variation Suite v8.6 *(Golden Helix*, *Inc*., *Bozeman*, *MT*, www.goldenhelix.com). Due to the use of feral and rare breeds we first evaluated the polymorphism content of the BeadChip transferable to these breeds. SNP with call rate <0.95 and located at sexual chromosomes were pruned from the initial dataset, leading to 57,668 SNP. Basic marker statistics as number of polymorphic SNP, average call rate and average number of alleles were calculated for each breed (Table S1).

The next step was remove markers not mapped in the Sscrofa10.2 assembly (2,674 SNP). The 54,994 remaining SNP were filtered using the following genetic parameters: Hardy-Weinberg Equilibrium (HWE) p < 0.0001, and Linkage Disequilibrium (LD) r^2^ > 0.3. The final data set had 8,764 SNP used in all autosomal analysis.

### Autosomal SNP Analysis

Within breeds genetic diversity parameters were estimated with 8,764 SNP using Arlequin 3.5.1.3^30^. Principal Component Analysis (PCA) using SNP & Variation Suite v8.6 (SVS) *(Golden Helix*, *Inc*., *Bozeman*, *MT*, www.goldenhelix.com) was performed to evaluate population stratification. The SVS software implements the EIGENSTRAT algorithm^31^. The results were represented in a 3D scatterplot of the first three principal components using SigmaPlot 13 (https://systatsoftware.com/products/sigmaplot/).

Population structure analysis was carried out using ADMIXTURE 1.2.2^31^ which is based on maximum likelihood. The inference of number of populations or clusters (K) present in the analysis was estimated by the cross-validation procedure., where the smallest cross-validation error rate indicates the most probable K value for the data set^33^. The default termination criteria for the ADMIXTURE analysis was assumed when the log-likelihood increases by less than ϵ = 10^−4^ between interactions. The termination criteria of is ϵ = 10^−5^ between interactions was also tested, without any difference on the cross-validation error values. The routine analysis was carried out for 22 clusters (Ks) with 10 replicates for each one. A second ADMIXTURE analysis with nine populations consisting of minor and rare breeds, and feral populations (12 clusters tested with 10 replicates each) was performed. The graphic representations of the proportions of assignment of each individual within population were visualized using CLUMPAK (http://clumpak.tau.ac.il/)^34^.

### Chromosome Y

The GGP Porcine HD v1 contains 14 SNP within the Chromosome Y. The analysis of the Y chromosome in mammals reflects the paternal history of migration and evolution of breeds and populations. For this analysis all female samples were eliminated, therefore 475 male samples were used. The 14 markers were pruned using a call rate <0.95. Of the remaining 11 SNP, two were monomorphic, and three more presented heterozygosity ≠ 0. Ultimately, six SNP were used to estimate the number of different haplotypes, haplotypes frequencies and to reconstruct a network among populations. The haplotype estimates and frequencies were calculated by the software Arlequin 3.5.1.3^30^ and the relationship between haplotypes was investigated by constructing median networks using Network 5 (http://www.fluxus-engineering.com/).

Effective Population Size: To establish baselines and trends of Ne the SNeP software^34^ was used with default conditions. Prior to using SNeP, SNP filtering was redone dropping the LD aspects. As a result 16,021 SNP were used in this analysis. Due to small sample sizes for some breeds the LD analysis was performed on 10 groups indicated by the population structure identified when ADMIXTURE analysis used K = 10. The 10 populations were: BE, CW, DU, a Chinese group (ME, FE, MI), a Pacific island group (GI, HI, KI), a minor breed group (GH, TA, OI, LB, SP, MA), HS, HE, LA, and YK^35^.

### Disclaimer

USDA, Agricultural Research Service, is an equal opportunity/affirmative action employer. Mention of companies or commercial products does not imply recommendation or endorsement by the USDA over others not mentioned. USDA neither guarantees nor warrants the standard of any product mentioned. Product names mentioned solely to report factually on available data and provide specific information.

## Supplementary information

Table S1 – Overall characteristics of the 18 populations genotyped with the SNP Beadchip after initial quality filter with a total of 57,668 SNPs.

## Data Availability

Molecular data and other information about the animals used in this study are available via the Animal-GRIN database: https://nrrc.ars.usda.gov/A-GRIN/genomic_account_page?record_source=US.
