# Supplementary material for: Assessing Sus scrofa diversity among continental United States, and Pacific islands populations using molecular markers from a gene banks collection
Source: Sci Rep. 2019 Feb 28;9:3173. doi: 10.1038/s41598-019-39309-9 (PMC6395668; doi:10.1038/s41598-019-39309-9)
Supplement: Supplementary file 1 — Table S1 – Overall characteristics of the 18 populations genotyped with the SNP Beadchip after initial quality filter with a total of 57,668 SNPs. [file 41598_2019_39309_MOESM1_ESM.pdf]

Assessing *Sus scrofa* diversity among continental United States, and Pacific islands populations using molecular markers from a gene banks collection

D. A. Faria[1,2], C. Wilson[2], Samuel Paiva[3,2], H. D. Blackburn[2*]

[2]National Animal Germplasm Program, National Center for Genetic Resources Preservation

ARS-USDA, Fort Collins, CO, 80521

Table S1 – Overall characteristics of the 18 populations genotyped with the SNP Beadchip after initial quality filter with a total of 57,668 SNPs.

Population	Code	N	# polymorphic	Average Call Rate	Average Number of Alleles	Average Ho
Berkshire	BE	43	52,934	0.995	1.917	0.285
Chester White	CW	27	53,691	0.997	1.931	0.337
Duroc	DU	64	49,203	0.994	1.853	0.286
Hereford	HF	22	48,397	0.997	1.839	0.291
Hampshire	HS	38	49,800	0.996	1.863	0.274
Yorkshire	YK	106	54,439	0.996	1.994	0.335
Landrace	LA	35	54,305	0.984	1.941	0.364
Mangalitsa	MA	3	26,415	0.984	1.458	0.225
Hawaii	HI + KI	34	53,555	0.927	1.928	0.285
Spotted	SP	10	49,625	0.997	1.860	0.317
Large Black	LB	3	30,675	0.974	1.531	0.250
Ossabaw Island	OI	5	33,266	0.993	1.576	0.252
Tamworth	TA	10	48,636	0.998	1.843	0.328
Guinea Hog	GH	4	32,416	0.967	1.562	0.271
Guam Island	GI	6	38,250	0.880	1.663	0.240
Meishan	ME	52	32,389	0.994	1.561	0.171
Fengjng	FE	19	33,893	0.994	1.587	0.191
Minzhu	MI	19	52,631	0.995	1.912	0.314
